# Gene electrotransfer with flow-through microchannel and lower alternating voltage generated induced pluripotent cells from human lymphoblastoid cell lines

**DOI:** 10.1371/journal.pone.0333491

**Published:** 2025-09-26

**Authors:** Miho Ishii-Teshima, Koki Maeda, Kazuki Hanauchi, Emika Asechi, Defan Setyawan, Takeshi Niki, Kenji Nakashima, Hirofumi Kurita, Rika Numano, Takayuki Shibata

**Affiliations:** 1 Department of Mechanical Engineering, Toyohashi University of Technology, Toyohashi, Japan; 2 Research Fellow of Japan Society for the Promotion of Science, Chiyoda, Tokyo, Japan; 3 Department of Applied Chemistry and Life Science, Toyohashi University of Technology, Toyohashi, Japan; 4 National Institute of Technology (Kosen), Sasebo College, Sasebo, Nagasaki, Japan; 5 Institute for Research on Next-generation Semiconductor and Sensing Science (IRES^2^), Toyohashi University of Technology, Toyohashi, Japan; Arizona State University, UNITED STATES OF AMERICA

## Abstract

Induced pluripotent stem cells (iPSCs) are useful for studying genetic and rare diseases and can be generated by reprogramming immortalized lymphoblastoid cell lines (LCLs) stored in global repositories with detailed genotype and phenotype data. Traditional bulk-type electroporators are commonly used for gene electrotransfer in reprogramming, but they have major drawbacks, including high costs associated with electric pulse generators and the requirement for fixed volumes for costly reprogramming factors. These limitations hinder cost-effective and scalable iPSC generation, particularly when working with large numbers of LCLs with diverse genotypes. We aimed to develop a flow-through-type electroporator utilizing microchannels for the generation of iPSCs from LCLs, to reduce the costs associated with traditional bulk-type electroporators and enable parallel processing for LCLs with various genotypes. We applied a continuous wave of biphasic alternating voltage (~10 V one-sided amplitude) to micro-scaled electrodes within the microchannel to develop a flow-through electroporator. Numerical simulations were conducted to assess the electric field distribution and its applicability to pore formation in the plasma membrane. To optimize electroporation and flow conditions, we used plasmid pCXLE-EGFP (encoding Green Fluorescent Protein, GFP) for gene electrotransfer to LCLs. Reprogramming factors (pCXLE-hSK, pCXLE-hOCT3/4-shp53-F, pCXLE-hUL) were also delivered to the cells via the same system. The flow-through electroporator achieved 31% transfection efficiency with 78% cell viability, 2 d post-electroporation. In each condition, only 3 µL of cell suspension was used with 10^7^ cells/mL of cells and 500 ng/µL plasmid vector. A reprogramming efficiency of 0.048% was obtained, which is comparable to that achieved using bulk-type electroporators. This developed flow-through electroporator with microchannel technology offers significant advantages over traditional methods, including the potential to reduce costs and the ability to process small volumes of cell suspension, making it suitable for parallel processing of LCLs with diverse genotypes. The system provides a promising approach for scalable and potentially cost-effective iPSC generation.

## Introduction

Human Epstein-Barr virus (EBV)-transformed lymphoblastoid cell lines (LCLs) are immortalized cell lines widely used in pharmacogenomic studies due to their easy sampling without biopsy, culturing, and storage. Large LCL collections are available worldwide [1], often with accompanying genotype and phenotype data. In the 2010s, researchers successfully generated induced pluripotent stem cells (iPSCs) from LCLs [2–6]. Notably, even after reprogramming, the EBNA-1 gene from EBV is not detected in the resulting iPSCs [3], which exhibit characteristics similar to iPSCs derived from fibroblasts. Fujimori et al. differentiated functional neurons from iPSCs generated from LCLs [5], facilitating drug development, disease modeling, and genetic disease research by leveraging patients’ genotype data available through repositories [5,7].

Conventional iPSC generation methods typically rely on viral vectors like retroviruses [8], which can integrate into the genome and cause mutations. Recently, non-viral methods using episomal plasmid vectors transferred by gene electrotransfer have gained popularity due to their lower mutation risk. Many protocols, such as those published by the Center for iPS Cell Research and Application (Kyoto University), now employ episomal plasmid vectors delivered by gene electrotransfer [9].

Gene electrotransfer, discovered by Neumann et al. in 1982 [10], involves suspending cells and DNAs in a cuvette with electrodes and applying an electric pulse. The pulsed electric field generated by the electric pulse induces a potential difference across the membrane, creating pores and membrane–DNA composites. This allows DNA molecules to be transferred into the cell nucleus [11,12]. The required membrane potential difference is approximately 200 mV [13]. For mammalian cells, an electric field of 0.1–1 kV/cm with a pulse duration of over 100 µs is required for effective gene electrotransfer [11,12]. Gene electrotransfer offers advantages such as reduced biohazard risk, no need for special chemicals, and lower dependency on cell type. This process of electric pore formation on the membrane, including gene electrotransfer, is known as electroporation, and various types of gene electroporation equipment, called electroporators, have been developed.

However, electroporators have two main disadvantages. First, they require high-voltage pulses. To achieve the 1 kV/cm threshold for pore formation, a 100 V pulse is needed across a 1 mm gap. This requirement makes the equipment large and cumbersome. High voltage also leads to electrolysis of the buffer and electrodes, resulting in bubble formation, metal particle release [14], and pH changes [15], all of which can reduce cell viability. Second, electroporators have fixed sample volumes, with commercial cuvettes typically holding 10–100 µL. To optimize transfer rates, testing is needed for each cell type to determine the appropriate voltage, pulse number, and duration. This results in wasted expensive plasmid vectors and cell samples due to fixed cuvette sizes.

We reported on droplet-electroporation, which uses aqueous microdroplets of less than 10 µL in oil as microreactors [16–20]. This approach reduces costs, as it only requires a direct current electric power source and a small amount of cell suspension. An oil-free method was also explored, where a small electrode pair positioned around the cell suspension enabled the processing of minimal volumes. Droplet-like application of electric stimuli was achieved using nanosecond-order short pulses [in preparation]. However, these methods still require high voltages in the kV range, and a fixed cell suspension volume is necessary for stable processing.

To address these limitations, we explored gene electrotransfer using flow-through microchannels. Various microchannel-based gene electrotransfer methods have been reported [21–34]. The smaller dimensions of microchannels and electrodes enable flow-through processing and create more intense electric fields with lower applied voltages. These systems are designed for specific gene electrotransfer applications, such as cell therapy with CRISPR/Cas9 [24,33]. Most studies focus on high-throughput strategies, processing several mL/min, though some are designed for individual processing or evaluations involving a small number of cells [34].

Although microchannel-based gene electrotransfer methods for mammalian cells have been widely studied, the use of these systems for episomal plasmid vector transfer to generate iPSCs has not been reported. This application faces three main challenges. First, it requires costly sample volumes. Liquid loading methods, such as syringe pumps and tubing systems, demand additional liquid to fill the tubing and syringe reservoir. Second, electroporation media are restricted. Most microchannel-based systems use low-conductivity media to prevent electrolysis-induced bubbling; however, LCL electroporation requires a culture medium with electrolytes [35]. Using a low-conductivity buffer can damage cell membranes [19,20]. Third, system manipulation is complex. Some microchannel systems need precise liquid control under microscopic observation, and often, multiple liquids require precise management, making them impractical for routine use.

In this study, we generated iPSCs from LCLs using a simplified microchannel-based gene electrotransfer method that requires only small volumes (3 and 12 µL per experiment) and high-density cell suspensions (10^7^ cells/mL). The required medium volume is lower than in bulk methods, yet sufficient cell numbers (10^4^ to 10^5^ cells per process) are obtained for culturing in 96- or 12-well plates. LCLs and plasmid vectors were suspended in Opti-MEM I and loaded into the microchannel with electrodes. A biphasic oscillating voltage with an amplitude of ~10 V was applied to the flowing sample, resulting in observed GFP gene expression. Furthermore, gene transfer of iPSC reprogramming factors was successfully performed, with reprogramming efficiency comparable to previous studies. This setup has the potential to improve the iPSC generating process and reduce associated cost. Furthermore, it can improve the gene electrotransfer process by integrating other microchannel-based systems, such as parallel processing, micro-mixing, cell sorting, dispensing, and culturing systems.

## Materials and methods

### Electroporator design

Our goal was to develop a microchannel-based electroporator with three main features: (1) continuous processing, (2) compatibility with standard conductivity media, and (3) user-friendly operation.

Traditional electroporators operate in batch mode, requiring a fixed sample volume in a cuvette, typically around 100 µL, which necessitates substantial cell, plasmid, and reagent quantities. However, our “continuous process” design removes sample volume restrictions, allowing for reduced sample and reagent use. The “no requirement of low conductivity medium” feature enables the use of standard buffers, including cell culture media with electrolytes, enhancing the device’s versatility. Finally, “easier manipulation” ensures that minimal setup time and optimization are needed, allowing for efficient handling of multiple experimental conditions.

**Fig 1:**
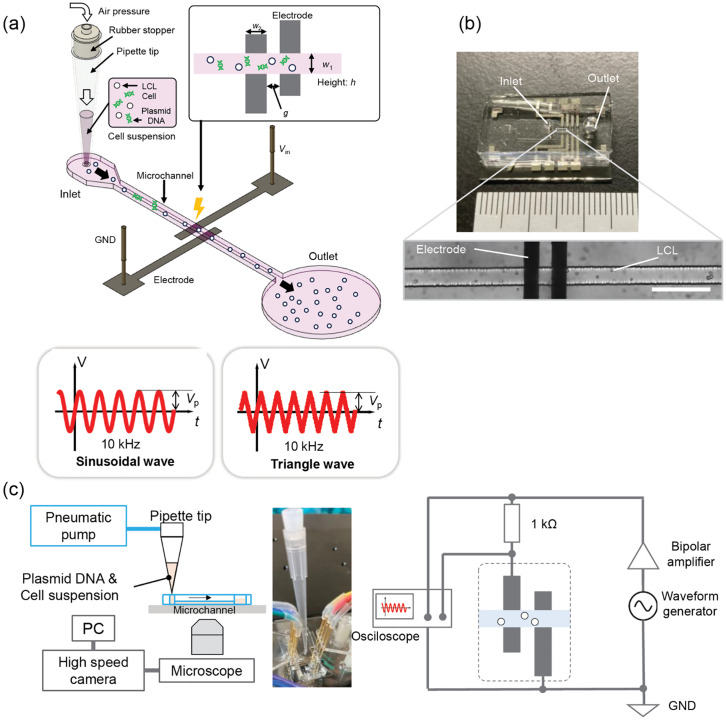
Experimental setup of gene electrotransfer with microchannel. (a) A schematic drawing of a microchannel chip for gene electrotransfer. Cells and plasmid vector suspension were introduced into single-lined microchannel via pipette tip and pneumatic pressure. An alternating biphasic wave, either sinusoidal or triangular, was applied across the electrodes, forming an electric field around them that facilitated plasmid vector transfer into cells. (b) Fabricated microchannel chip with inlet and outlet biopsy punches of 1- and 3-mm diameter, respectively. An unused branch channel for droplet formation was also visible [36]. The scale bar is 200 µm. (c) Experimental setup for flow generation and voltage application. A pneumatic pump (Flow EZ 7000 mbar, Fluigent) was attached to the pipette tip at the inlet. The electric power source consisted of a waveform generator (33500B, Agilent) connected to a bipolar amplifier (BA4825, NF Co.). A 1 kΩ resistor was connected to the electrodes, and an oscilloscope (PicoScope 6404E, P2056, Pico Technology) monitored the electrode voltage. The voltage amplitude decreased as the solution contacts the electrodes, and amplitude fluctuations indicated cell passage, which helped to monitor channel activity.

The electroporator system incorporates a PDMS-based microchannel with electrodes and an alternating voltage application. Fig 1(a) provides a schematic of the electroporation setup. The device includes electrode pairs positioned within a single microchannel, into which cell and plasmid suspensions are introduced directly through a micropipette tip connected to the channel inlet. Pneumatic pressure applied to the pipette tip induces liquid flow, causing samples to pass through the electrode region where an alternating voltage generates an intense electric field. As cells pass through this field, gene transfer occurs.

Following electroporation, processed cells exit through the outlet and are collected with a micropipette for transfer to the cell culture medium. This “continuous process” design with microchannel allows samples exceeding the channel’s internal volume (~9 nL) by using the pipette tip as an adaptable inlet reservoir [37]. The maximum sample volume depends on the pipette’s capacity, though excessive volumes may cause cell sedimentation and clogging due to prolonged processing times. For this study, single-process sample volumes were 3 µL for GFP-coding plasmid vectors and 12 µL for iPSC reprogramming factor.

### Numerical simulation of electric field

Pore formation in the plasma membrane by transmembrane voltage is known to initiate gene electrotransfer, although the process also depends on physiological mechanisms [12,38]. To confirm whether the electric field generated in the microchannel could induce cell permeabilization, we performed numerical simulations of the electric field and pore formation using COMSOL Multiphysics ver. 6.2 (https://www.comsol.com/).

The microchannel was modeled with dimensions of 50 µm width, 30 µm height, and 200 µm length. An electrode pair was positioned at the bottom of the channel, with a 40 µm gap and 50 µm electrode width. A 10 V potential was applied to the left electrode, with the right electrode connected to the ground. Two models were simulated: one without cells, where only the medium filled the channel, and one with a cell present. The buffer conductivity was set at 1.38 S/m, measured using a LAQUA act ES-71 with a conductivity meter (HORIBA Co.) in Opti-MEM I. For the model with a cell, a spherical cell with a 12 ± 2 µm diameter (as measured by the optical microscopy of LCLs) was placed at the center of the electrode gap within the channel. Additional parameter values were taken from the literature, as shown in Table A in S1 Appendix [39–42]. The total element count for the simulations was 255,504 and 274,050 for channels without and with a cell, respectively.

Calculations were based on the current conservation equation, incorporating membrane lysis using the asymptotic Smoluchowski equation, as outlined by Abdelmoez et al. [43]. Details of the calculation setup are provided in Fig A in S1 Appendix. The simulations did not account for the formation of electric double layers, surface chemical reactions at the electrodes, or specific physiological reactions of membrane proteins, such as proton pumps.

To simplify the calculations, direct current was used in the simulation to represent the applied voltage. Although our experimental setup involved a 10 kHz oscillating voltage, the time response of membrane potential could be approximated using Schwan’s equation for transient responses [44],

$\Delta V=\frac{\frac{\text{3}}{\text{2}}\;E\;R\;\text{cos}\;\theta }{\text{1}+i\omega RC_{\text{m}}\left(\rho_{\text{i}}+\frac{\text{1}}{\text{2}}\;\rho_{\text{a}}\right)}$

where *E* is the electric field, *R* is the cell radius, *θ* is the angle on membrane, *f* is the frequency of the electric field, *C*_m_ is the membrane capacitance, and ρ_i_ and ρ_a_ represent the resistivity of cytoplasm and medium, respectively. The time constant for transmembrane potential, τ, is given by:


$$\tau =RC_{\text{m}}\left(\rho_{\text{i}}+\frac{\text{1}}{\text{2}}\;\rho_{\text{a}}\right)$$

For a cell with a 12 µm diameter and a membrane capacitance *C*_m _= 1.0 × 10^−2^ F/m^2^ [45], along with cytoplasm resistivity *ρ*_i_ = 1.25 Ω·m [43] and Opti-MEM I medium resistivity *ρ*_a_ = 0.724 Ω·m, the calculated time constant is τ = 1.9 × 10^−7^ s. This is three orders of magnitude smaller than 0.1 ms, the period of a 10-kHz waveform. Therefore, based on these estimates, direct current simulation is appropriate for representing the electric field in this system.

### Fabrication

The microchannel was designed with a width of *w*_1_ = 50 µm, height *h *= 30 µm, and length *L* = 5.8 mm, which is sufficient to allow cells, with a mean diameter of 12 ± 2 µm, to pass through. Fig 1 (b) shows the fabricated microchannel chip, which was created by soft lithography using PDMS and bonded to a glass substrate via plasma bonding (Fig B in S1 Appendix). Actual measurements of the channel dimensions were *w*_1 _= 52 ± 2 µm and *h* = 27 ± 1 µm (*n* = 5), with the cross-sectional shape slightly tapered rather than a perfect rectangle (Fig C in S1 Appendix).

Each substrate contained four electrode pairs, although only one pair was used per single process. Electrodes were fabricated using a lift-off process, with photoresist and subsequent sputtering of Pt over a Ti layer. The Pt and Ti layers were 100 and 10 nm thick, respectively. The designed electrode dimensions were *w*_2 _= 50 µm (53 ± 3 µm, *n* = 32) for electrode width and *g* = 40 µm (40 ± 3 µm, *n *= 16) for the gap. Measurements obtained using a high-speed camera (HAS-D71G, Ditect) confirmed electrode dimensions as *w*_2 _= 53 ± 3 µm (*n* = 32) and *g* = 40 ± 3 µm (*n *= 16).

### Gene electrotransfer with microchannel

Fig 1(c) shows the experimental setup. The microchannel chip was mounted, with the pipette tip containing sample liquid directly inserted into the inlet, followed by the removal of the micropipette. Pneumatic pressure was applied through the micropipette to accelerate the liquid flow. The electric power source applied a 10-kHz biphasic voltage wave with sinusoidal or triangle waveform on the electrode pair.

The flow within the microchannel was visualized using an inverted microscope (Eclipse Ti2-U, Nikon) and a high-speed camera (HAS-D71G, Ditect). Extracted liquid flowed into the punched outlet. After processing, approximately 10 µL of Opti-MEM I was added to the outlet, mixed by pipetting, and then collected for transfer to the cell culture medium. Each process took about 1 min for a 3-µL sample and 3 min for a 12-µL sample.

### Cell preparation

In this study, human B lymphocytes transformed by the Epstein-Barr virus (HEV0019) [46] obtained from Riken BRC with 25 passage generation, were used for the experiment. Cells were cultured in RPMI1640 medium (189–02025, Wako) with 10% fetal bovine serum (CCP-FBS-BR-500, Cosmo Bio) and 1% penicillin-streptomycin (15140122, Gibco). Cells were seeded in 5 mL of cell culture medium.

Every 2–3 days, cells were centrifuged at 200 ×* g* for 3 min. The supernatant was removed, and fresh culture medium was added to achieve a seeding density of 5 × 10^5^ cells/mL. The cell culture dish was incubated under 5% CO_2_ and 37°C. Before experiments, cells were centrifuged again at 200 ×* g* for 3 min and suspended in 1 mL Opti-MEM I reduced serum medium (31985070, Gibco) to prevent cell aggregation. After passing the suspension through a strainer, the supernatant was removed, and 105 µL of Opti-MEM I was added. A 5-µL sample was taken for cell counting, with Trypan blue used to stain dead cells. Finally, Opti-MEM I and plasmid were added to achieve the desired final cell density.

### Transfection with pCXLE-EGFP plasmid vector

The episomal plasmid vector pCXLE-EGFP, used to express GFP, was gifted by Shinya Yamanaka of Addgene (Addgene plasmid # 27082; http://n2t.net/addgene:27082) [47]. For gene electrotransfer, we suspended LCLs and the plasmid vector in Opti-MEM I, with cell and plasmid concentrations of 10^7^ cells/mL and 500 ng/µL, respectively. As a negative control (NC), we added 2 µL of this cell and plasmid suspension to 100 µL of cell culture medium in a 96-well plate without electroporation processing.

For each gene electrotransfer experiment, 3 µL of the prepared cell and plasmid suspension was pipetted directly into the microchannel. The electrotransfer process was performed as mentioned above. After electroporation, treated cells were added to the 100 µL of cell culture medium in a 96-well plate and incubated for 2 days.

Post-incubation, cells were stained with 9 µM PI solution (red fluorescence dye; P378, Dojindo) for dead cell detection and 10 µM Hoechst 33342 solution (blue fluorescence dye; H342, Dojindo) for whole cell staining. Hoechst 33342 is generally used for living cell staining; however, in our measurement method, the dead cells with weak fluorescence of Hoechst 33342 tended to be judged as positive. Approximately 15 min post staining, cells were imaged on an inverted fluorescent microscope (Eclipse Ti2-U, Nikon) equipped with optical filters (FITC C-FLL-C for green fluorescence, mCherry C-FLL-C for red fluorescence, DAPI C-FLL-C for blue fluorescence, Nikon) and a color camera (DS-Ri2, Nikon). A 20x objective lens (CFI S Plan Fluor DLWD ADM 20xC, Nikon) was used for detailed observation, while a 4x objective (CFI Plan Fluor DL 4x, Nikon) was used for cell counting.

ImageJ software was used for cell counting, with manual adjustments based on supporting image processing protocols (Supporting Figs D and E in S1 Appendix). Counts for GFP-positive cells (*n*_GFP_), Hoechst 33342-positive cells (*n*_Hoechst_), and PI-positive cells (*n*_PI_) were recorded for both experimental and NC samples (*n*_GFP,NC_; *n*_Hoechst,NC_; and *n*_PI,NC_). Based on prior research [26 29 48], transfection efficiency was calculated as:


$$(\text{Transfection efficiency})=\frac{n_{\text{GFP}}}{n_{\text{Hoechst}}-n_{\text{PI}}}$$

Cell viability was determined as:


$$(\text{Cell viabliity})=\text{1}-\left(\frac{n_{\text{PI}}}{n_{\text{Hoechst}}}-\frac{n_{\text{PI},\text{NC}}}{n_{\text{Hoechst},\text{NC}}}\right)$$

Given the floating nature of LCLs, separating dead cells prior to the processing was challenging. Dead cell counts varied by culture conditions; hence, NC values were subtracted from post-processing results to control for initial cell death variability.

### iPSC generation

Induced pluripotent stem cell generation was achieved using episomal plasmid vectors—pCXLE-hSK, pCXLE-hOCT3/4-shp53-F, and pCXLE-hUL—selected for high reprogramming efficiency [47]. Each vector was prepared at a concentration of 167 ng/µL, totaling 500 ng/µL plasmid DNA concentration, equivalent to the GFP plasmid used in earlier transfection experiments. Cells were suspended at a density of 10^7^ cells/mL, and 12 µL of the solution was pipetted into the microchannel. After electrotransfer, the processed cells were seeded into 12-well plates pre-treated with imatrix-511 silk, a substrate for cell culture, with each well containing 700 µL of LCL culture medium.

For cell maintenance, 700 µL of Stemfit medium (StemFit AK02N, REPROCELL) was added on days 2, 4, and 6 post-processing. From days 8–21, the medium was replaced every two days with 1 mL of fresh Stemfit medium. After 21 days, cells were observed via bright-field microscopy, then fixed with 500 µL of 4% paraformaldehyde phosphate buffer solution (166−23555, FUJIFILM Wako) following two PBS washes. Pluripotency was evaluated using the Leukocyte Alkaline Phosphatase Kit (85L1-1KT, Sigma-Aldrich), where 48 mL of Fast blue RR salt solution was prepared with Mili-Q water and combined with 2mL of naphthol AS-MX phosphate. A 1 mL aliquot of this solution was added to each well and stored for 30–60 min. Finally, the solution was removed and the wells were washed with PBS. Pluripotent colonies were visualized by PBS washing and manually counted via scanner (GT-X830, EPSON Co.). Reprogramming efficiency was calculated as the percentage of colonies formed out of the initial processed cell count (12 µL × 10^7^ cells/mL = 1.2 × 10^5^ cells).

### Statistical analysis

All data were presented as average values, with error bars and ± symbols indicating standard deviation. Statistical significance was assessed with two-tailed Student’s t-tests, with *p*-values designated as follows: *p* < 0.05, *p* < 0.01, p < 0.001 described as *,**, *** or #, ##, ###, respectively. In each experiment, cell velocity was measured from high-speed video recordings by randomly selecting 20 cells and calculating velocity based on two positions and time intervals. The typical sample standard deviation in velocity measurements was approximately 10%.

For fluorescent-based cell counting, only datasets with >1000 Hoechst 33342 stained cells were used for accuracy. Cell counts varied based on fluid handling (e.g., inlet suspension residuals or pneumatic pressure at the outlet) and culture conditions, which could impact cell proliferation. Detailed raw data for each experimental condition are presented in Tables B–G in S1 Appendix.

### Ethics statement

The data of cell sample obtained from Riken BRC were completely anonymized, with no personally identifiable information accessible to the researchers.

This study involved gene transfer into human cells and the establishment of iPSC. However, no transplantation of the generated iPS cells into living organisms was performed. Therefore, the study did not fall under the scope of regulations concerning genetically modified organisms as defined by the Cartagena Act.

## Results

### Numerical simulation of electric field

**Fig 2:**
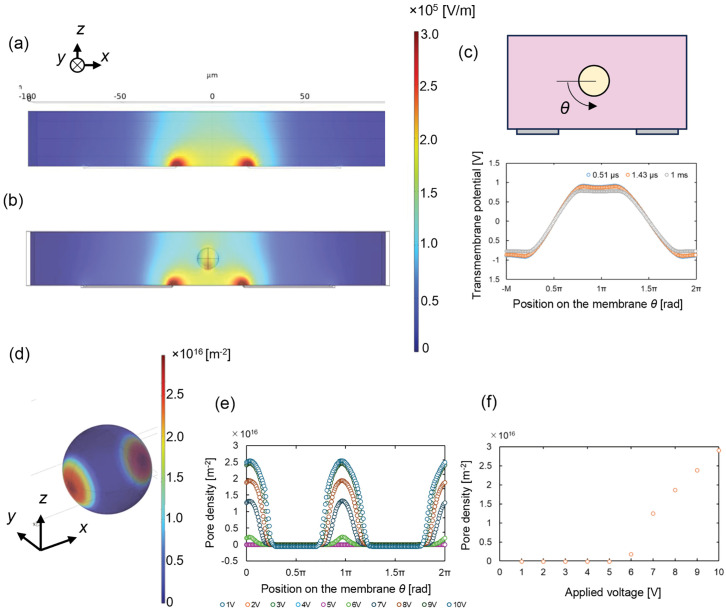
Results of numerical simulation of the electric field in the microchannel under a 10-V application. (a) Intensity of electric field in the microchannel without cells. (b) Intensity of electric field when a cell is centered in the channel between electrodes at 1 ms. (c) Transmembrane potential distribution on cell membrane at 1 ms based on asymptotic Smoluchowski equations. (d) Distribution of pore density on the plasma membrane. (e) Distribution of pore density on plasma membrane circumference at various application voltages at a time of 1 ms. (f) Pore density at *q* = 0, and 1 ms in various applications.

Fig 2(a) illustrates the electric field intensity distribution in the microchannel. An electric field greater than 1 kV/cm was generated above the electrode pair, with high intensity concentrated between the inner edges of the electrode pair, while the field intensity diminished near the outer edges. This suggests that cells experience the primary electric stimulus as they passed through this central region of the electrodes.

Due to the planar placement of the electrodes at the bottom of the channel, the electric field was asymmetrically distributed along the z-axis. Although field intensity decreased toward the top of the channel, it remained above 1 kV, which was sufficient for effective electroporation. However, at the channel’s bottom near the electrode edges, a highly localized intense electric field was observed, potentially leading to cell damage or death.

As the cell interacted with the electric field, a transmembrane voltage formed due to the plasma membrane’s capacitance, resulting in a localized perturbation in the electric field (Fig 2 (b)). However, the field’s high-intensity regions remain consistent with those in Fig 2(a), indicating minimal disturbance from the cell and suggesting that cell-to-cell interactions in the field were minimal.

Transmembrane potential on the plasma membrane is shown in Fig 2(c). The highest potential was concentrated at both polar regions adjacent to the electrodes, reaching approximately 0.8 V. The peak potential flattened due to voltage drop from pore formation, as described by the asymptotic Smoluchowski equation.

Pore density distribution is shown in Fig 2(d). Electrically formed pores were located on the cell membrane in a circular-shaped area. Fig 2(d) shows the pore density distribution when applying 1–10 V. Pore formation occurred around *θ* = 0.03 π and 0.97 π when voltage above 6 V was applied. At 5 V, the pore density dropped by more than two orders of magnitude, falling below 10^13^ m^-2^. Fig 2(e) shows pore density relative to the applied voltage, with ~6V appearing to be the threshold required for effective pore formation.

### Gene electrotransfer of pCXLE-EGFP plasmid vector

To optimize the experimental parameters, gene electrotransfer of the pCXLE-EGFP plasmid vector was conducted with varying voltage while maintaining a fixed pneumatic pressure of 10 kPa. Sinusoidal and triangle biphasic waveforms at 10 kHz frequency were used. The average cell velocity was measured at 61 ± 12 mm/s (*n *= 57), resulting in an effective electric exposure time of approximately 0.66 ms as cells passed through the 40 µm electrode gap. This duration corresponded to around 6.6 cycles of the oscillating field per cell pass.

**Fig 3:**
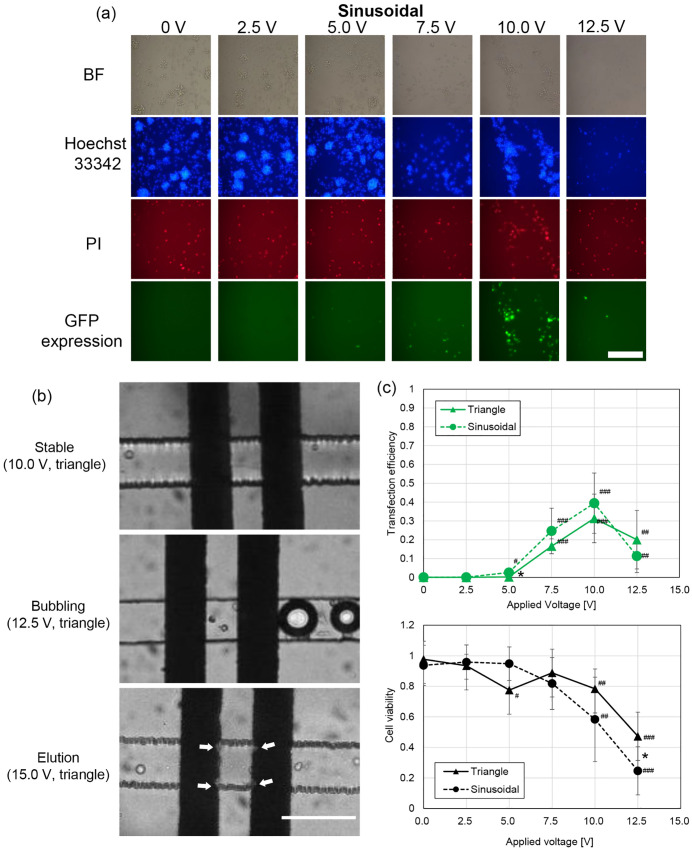
Experimental results of gene electrotransfer with pCXLE-EGFP plasmid vector as a parameter of applied voltage. (a) Images of LCLs with bright field (BF), the fluorescence of Hoechst 33342 (blue), PI (red), and GFP expression (green). Bright-field images and fluorescent-filtered images with the filters of FITC, mCherry, and DAPI are taken, respectively. Positions of cells were slightly moved while taking with each fluorescent filter because LCLs are floating cells. Scale bar = 200 µm. (b) Microscopic images of microchannel around electrode pair. White arrows show the location of electrode elution. Bubbling and elution of electrodes are sometimes observed above 12.5 V application. Scale bar = 100 µm. (c) Transfection efficiency and viability of LCLs as a parameter of application voltages for sinusoidal wave and triangle wave. Significant differences between 0V application and other voltages are indicated by #. Significant differences between triangle and sinusoidal waves are depicted by *.

Fluorescence microscopy images of LCLs under each voltage condition are shown in Fig 3(a), with GFP expressions observed at voltages exceeding 5V. As voltage increased, a higher proportion of dead cells were noted. At voltages above 12.5 V, small bubbles occasionally appeared along the electrode edges. Although bubbling ceased after a few seconds, voltages of 15 V caused considerable bubbling, cell fragment deposits on electrodes, and electrode degradation (Fig 3(b)). Hence, the voltage application range was limited to 12.5 V in this system.

The transfection efficiency of GFP expression and cell viability results are shown in Fig 3(c), showing similar responses to voltage across both sinusoidal and triangle waveforms. At lower voltages (*V*_p_ = 2.5 and 5.0 V), transfection efficiency was low. However, at *V*_p_ values of 7.5 and 10.0 V, both higher transfection efficiency (>10%) and cell viability of >50% were achieved. The estimated threshold for effective gene electrotransfer lies between 5.0 and 7.5 V, with *V*_p_ = 10 V producing the highest GFP expression. At *V*_p_ = 12.5 V, cell death increased notably, and transfection efficiency decreased compared to 10 V. A lower viability observed with 5V in the triangle waveform condition resulted from cell handling and installation issues rather than the electrical parameters. Variability in cell density bias in the suspension likely contributed to lower viability in a single experiment.

In general, transfection efficiency tended to be higher with the sinusoidal waveform than with the triangle waveform, though the difference was not statistically significant (*p* > 0.05) except at *V*_p_ = 5 V. For cell viability, a trend of higher viability was observed with the triangle waveform, particularly at 12.5 V, where the difference was statistically significant.

**Fig 4:**
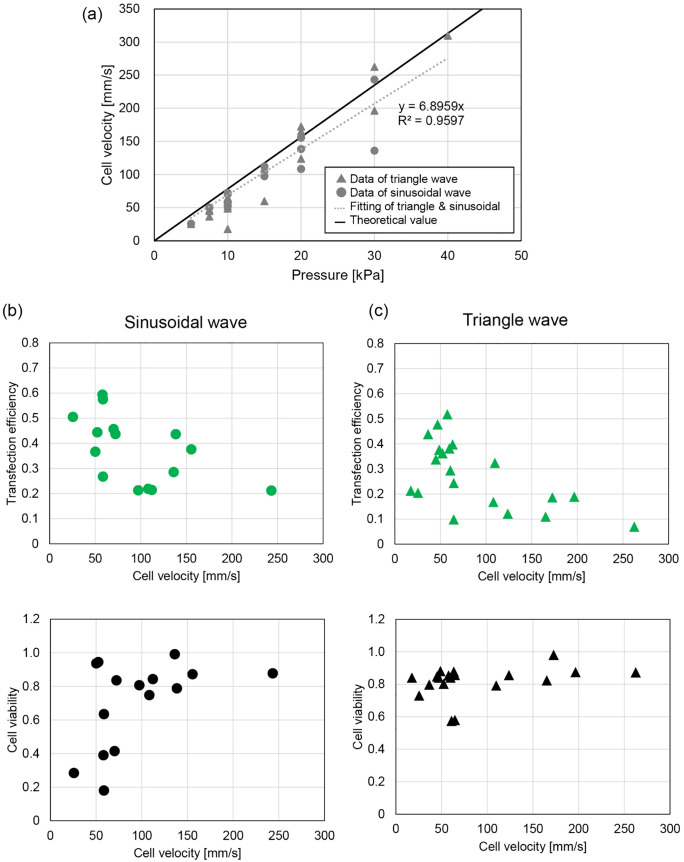
Experimental results of gene electrotransfer with pCXLE-EGFP plasmid vector as a parameter of cell velocity. (a) Measured cell velocity in each applied pneumatic pressure. The theoretical value was calculated based on the flow speed of the rectangular channel [49]. Transfection efficiency and cell viability as a parameter of flow velocity for (b) sinusoidal wave and (c) triangle wave. *V*_in_ = 10 V was applied in each experiment.

To further investigate the influence of flow speed, pneumatic pressure was varied from 5 to 40 kPa, with an applied voltage of *V*_p_ = 10 V. Fig 4(a) presents the cell velocity under each applied pressure. Theoretical velocity was calculated using flow dynamics for a rectangle channel [49],

$v=\frac{pH^{\text{2}}}{\text{1}\text{2}\eta L}\left(\text{1}-\text{0}.\text{6}\text{3}\text{0}\frac{H}{W}\right)$

where *p* is applied pressure, *H* is channel height, *W* is channel width, *L* is the channel length, and η the viscosity. Using measured channel dimensions *H *= 27 µm, *W *= 51 µm, *L* = 5.8 mm, with viscosity η = 0.9 mPa ⋅ s (based on water at 25°C), the observed cell velocities closely matched theoretical values.

The measured cells velocities in each attempt are shown in Fig 4(b) (c), displaying the relationship between velocity, transfection efficiency, and cell viability. For the sinusoidal waveform, maximum transfection efficiency reached 59% with a viability rate of 39%, while for the triangle waveform, the maximum efficiency was 52% with a viability rate of 85%. Both waveforms achieved high transfection efficiencies within a cell velocity range of approximately 50–100 mm/s. However, a notable difference in viability between the two waveforms was observed. At slower cell velocities, the sinusoidal waveform led to higher cell death compared to the triangle waveform, likely due to the increased energy delivered by the sinusoidal wave, which caused more cellular damage.

As cell velocity increased, transfection efficiencies decreased for both waveforms, especially for the triangle waveform. This observation suggests that the triangle waveform might have required more cycles of the electric field to achieve effective gene transfer. Consequently, cells moving faster through the electric field might not have experienced sufficient exposure time to facilitate optimal transfection, particularly under the triangle waveform condition.

### iPSC generation

**Fig 5:**
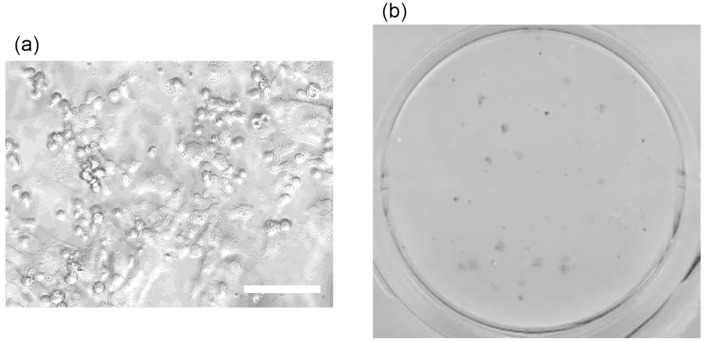
Images of generated iPSCs reprogrammed by gene electrotransfer to LCLs. (a) Microscopy images of iPSCs 21 days after gene electrotransfer. Flat-shaped cells are reprogrammed iPSCs, while sphererical cells are LCLs. Electrotransfer was performed using a triangle waverform at *V*_p _= 12.5 V with an air pressure of 10 kPa for acceleration. Scale bar = 100 µm. (b) Image of a 12-well plates containing cultured and fixed iPSCs. Alkaline-phosphatase-stained cell colonies are depicted as black points.

Gene electrotransfer was applied for iPSC reprogramming using various voltages, waveforms, and pneumatic pressures, with results summarized in Table 1. Microscopy images in Fig 5(a) show reprogrammed iPSC colonies, while Fig 5(b) displays stained colonies, indicating pluripotency. The number of data points was fewer for the 12.5 V with 20 kPa conditions than for other test cases. One experiment under this condition could not be completed successfully due to channel clogging with cells, which limited the number of experimental replicates.

**Table 1:** Experimental conditions and results of iPSC reprogramming.

Voltage V	Waveform	Pressure kPa	Velocity mm/s	Applied cycles of wave	Number of colonies	Reprogramming efficiency %	Number of data
10.0	Triangle	10	60.2 ± 0.8	6.6	33 ± 15	0.028	*n* = 3
10.0	Sinusoidal	10	60.7 ± 0.3	6.6	30 ± 16	0.025	*n* = 3
12.5	Triangle	10	51.3 ± 9	7.8	57 ± 6	0.048	*n* = 3
12.5	Sinusoidal	10	59.0 ± 2.6	6.8	11 ± 11	0.009	*n* = 3
12.5	Sinusoidal	20	141 ± 11	2.8	52	0.043	*n* = 2

Reprogramming efficiencies exceeded 0.02% under most conditions, except for the sinusoidal waveform at 12.5 V and 10 kPa, which showed a notably lower efficiency. At 10.0 V, both sinusoidal and triangle waveforms achieved similar reprogramming efficiencies. However, a clear difference emerged at 12.5V: the triangle waveform yielded the highest efficiency at 0.048%, while the sinusoidal waveform showed considerably lower efficiency under low pressure (slow cell velocity) conditions. Notably, at higher cell velocities, the sinusoidal waveform with 12.5 V demonstrated improved reprogramming efficiency, suggesting that faster flow rates may mitigate potential damage from prolonged electric exposure in sinusoidal conditions.

The iPSC generation experiment revealed that the sinusoidal and triangle waveforms performed differently based on specific conditions, reflecting trends similar to those observed with the pCXLE-EGFP plasmid transfection efficiency and cell viability results. As shown in Fig 3(c), the sinusoidal wave caused more cell death at higher voltages, potentially explaining the reduced reprogramming efficiency observed with 12.5 V sinusoidal wave at 10 kPa (0.009%). However, as shown in Fig 4(b), increasing cell velocity through higher pneumatic pressure (20 kPa) improved reprogramming efficiency for the sinusoidal waveform at 12.5 V to 0.043%, comparable to the triangle wave’s maximum of 0.048%. These findings suggest that adjusting cell velocity can mitigate cell death in sinusoidal conditions, allowing higher reprogramming efficiency.

## Discussion

Our goal was to develop an electroporator that facilitates “continuous process,” “compatibility with standard conductivity media,” and “easier manipulation.” Several microchannel-based electroporators were previously reported, each with advantages and disadvantages.

A common technique in microchannel electroporation relies on constrictions within the channel path [21,23,24,26,28]. In this design, the channel contains a wide section and a constricted region, with electrodes positioned at the start and end. The electric field lines concentrate at the constriction, creating an intense electric field. As cells pass through the constriction, they are exposed to a pulse-liked electric field. This method enables gene electrotransfer at higher flow rates.

However, this method has three main limitations. First, the power source requirement: these systems require relatively high voltages due to longer channel length, with power sources typically in the range of several tens of volts to over 100 V. Second, the need for a specific processing medium: only low-conductivity media can be used to reduce current, avoiding problems such as bubbling at the electrodes, temperature rise, and pH fluctuations. While low conductivity buffers are commonly used in electroporation, including bulk-type methods, some studies have successfully employed buffers similar to cell culture media, such as Opti-MEM I [35,50–52]. The third limitation is the relatively large amount of sample liquid required, as longer and wider channels necessitate a greater volume of sample fluid.

Placing electrodes with a narrow gap in the microchannel path can also generate a locally intense electric field within the channel [22,25,27,31,33,36]. The shape of this type of channel can be easily adjusted, and a small amount of sample liquid can be processed when using a narrow and short channel. To perform gene electrotransfer at lower voltages, we adopted this type of design.

However, this type of equipment faces challenges related to electrolysis at the electrodes. Electrolysis can cause bubbling, leading to channel clogging, cell damage, and changes in the pH of the buffer. Several strategies have been proposed to mitigate these issues. They include applying a voltage below the redox potential [27], separating the electrode and cell suspension using a gel [22], and separating the flow of the cell suspension from the flow at the electrodes to prevent mixing of cells with lysed buffer [25,31,33]. We previously attempted to apply an intense electric field while minimizing electrolysis using microdroplets [36]. However, these methods often result in more complex microchannel designs and require more complicated manipulations and adjustments.

Another approach to avoid electrolysis is the use of alternating voltage applications [23,29,30 32]. By applying a kHz-ordered bipolar alternating current, the amount of electrolysis in the channel can be reduced [23]. We adopted this approach of biphasic alternating voltage application in our system.

In direct current electroporation, the electric current between the electrodes and the solution is driven by electron exchange through chemical reactions. In contrast, with alternating voltage application, the reduction of bubbles is attributed to the charging time of the capacitance at the interface between the electrodes and the liquid, as well as the formation of nanobubbles. The voltage applied to the electrodes forms an electric double layer at the electrode-liquid boundary, which behaves as a capacitance in the circuit. During the capacitance charging, ions flow as current in the channel without undergoing a chemical reaction.

Furthermore, the rapid change in polarity results in the formation of nanobubbles, which are mixed reaction products. In the case of balanced alternating voltage application, the anode and cathode alternate, and the generated gas species mix in a stoichiometrically balanced ratio. The internal pressure of the nanoscale bubbles is high because Laplace pressure is inversely proportional to the boundary curvature of the bubble. This high pressure can cause the combustion of the gas species [53]. In literature, experiments on electrolysis with water and alternating voltage showed that the amount of bubbling was small under kHz-ordered frequencies. The researchers concluded that H_2_ and O_2_ at the electrodes combusted, and H_2_O was re-generated [53]. In our system, we assume that H_2_ and Cl_2_ are generated due to electrolysis, as the buffer contains Na^+^ and Cl^-^ ions. In the nanobubbles, these gases react and produce HCl, which is soluble in water. The observed result of “small amount of bubble is generated on the electrodes at the beginning of voltage application” in 12.5 V may be due to bubble nuclei forming on the electrodes.

Gene electrotransfer was successfully performed using our setup. The threshold voltage for gene electrotransfer was approximately 5 V, with higher efficiency observed at voltages of 7.5 and 10 V. In numerical simulations, the threshold for pore formation was estimated to be around 6 V, which corresponds well with the experimental results. Notably, at 12.5 V, the gene expression was lower compared to the 10 V setting. However, higher voltage application generally results in greater transfection efficiency but also increased cell death. The lower transfection efficiency at 12.5 V may be attributed to cell loss during the process, particularly from cells being exposed to the electric field before gene expression could occur. Additionally, the tapered cross-sectional shape of the channel may contribute to a lower electric field in certain areas, leading to some cells slipping through without being exposed to sufficient electrical stimuli.

The maximum transfection efficiency observed was 39% with viability of 58% using a sinusoidal waveform, and 31% with viability of 78% using a triangular wave. These values were lower than the efficiency achieved with commercial bulk-typed electroporators (>90%) and other microchannel-based electroporators (~75% for plasmid [24], 95% for mRNA [33]). The lower transfection efficiency in our system is likely due to the nature of LCLs, which are considered “hard-to-transfect cells” compared to other cell lines [35,54]. For example, a transfection efficiency of 79% with 58% viability (48 h after processing) has been reported for LCLs using a bulk-type electroporator and pCMV-GFP plasmid. Despite the low gene expression efficiency in our system, the damage to cells is lower with the triangular waveform compared to the bulk electroporation method. Another contributing factor to the lower efficiency may be the heterogeneous electric field produced by the electrodes. Since we used printed electrodes on a single substrate, the electric field varied in height, leading to non-uniform electric stimulation (as shown in Fig 2). We adopted this design for easier fabrication, but using parallel electrodes or focusing the cell path with sheath flow [55] could improve the dispersion of electric stimuli.

In iPSC generation, even if all the reprogramming factors are introduced, not all of the cells undergo reprogramming. Reprogramming efficiency for LCLs has been reported to range from 0.002% to 0.01% when using a transfection efficiency of 60% [2]. Previous studies have reported reprogramming efficiencies of 0.0054–0.0216% [6], 0.0015% and 0.002% [5], 0.001–0.06% [4], and 0.03% [47], typically within the range of 0.001 to 0.01%. Notably, reprogramming efficiency can depend on the type of cell line and plasmid vector used. Our results fall within this range, suggesting that the efficiency of our system is of a similar order for iPSC generation.

## Conclusion

We generated iPSCs from LCLs using a microchannel-based electroporation system designed for continuous processing, low conductivity buffer flexibility, and simpler manipulation. Gene electrotransfer was achieved with micro-gapped electrodes and an alternating current under 15 V with 10 kHz oscillation. Optimal conditions for gene transfer were determined using the pCXLE-EGFP plasmid vector. A voltage range of 7.5–12.5 V allowed for efficient gene transfer without clogging or bubbling, with a maximum transfection efficiency of 39% at 10 V and a passage time of 0.66 ms. A triangle waveform achieved 31% transfection efficiency with 78% viability concurrently. In iPSC generation, a maximum reprogramming efficiency of 0.048% was obtained, comparable to results from bulk electroporators.

This continuous microchannel-based system enables the processing of smaller cell suspensions, reducing the need for reagents and cells. Its low voltage requirement (< 15 V) is readily attainable with commercial ICs, offering a safer alternating to high-voltage pulses. Sample loading via pipette tip and pneumatic pressure also simplifies handling. These features may support a cost-effective gene electrotransfer setup for iPSC generation from LCLs.

## Supporting information

S1 Appendix Technical information and measurement data. (PDF)
