# Correction: Gene electrotransfer with flow-through microchannel and lower alternating voltage generated induced pluripotent cells from human lymphoblastoid cell lines

**DOI:** 10.1371/journal.pone.0339487

**Published:** 2025-12-22

**Authors:** Miho Ishii-Teshima, Koki Maeda, Kazuki Hanauchi, Emika Asechi, Defan Setyawan, Takeshi Niki, Kenji Nakashima, Hirofumi Kurita, Rika Numano, Takayuki Shibata

In the Funding section, the grant number from the funder JSPS KAKENHI is listed incorrectly. The correct grant number is: 22KJ1632.
