# Supplementary material for: Gene electrotransfer with flow-through microchannel and lower alternating voltage generated induced pluripotent cells from human lymphoblastoid cell lines
Source: PLoS One. 2025 Sep 26;20(9):e0333491. doi: 10.1371/journal.pone.0333491 (PMC12468887; doi:10.1371/journal.pone.0333491)
Supplement: S1 Appendix — (PDF) [file pone.0333491.s001.pdf]

Supporting information

Gene electrotransfer with flow-through microchannel and lower alternative voltage generated induced pluripotent cells from Human Lymphoblastoid cell lines

Miho Teshima-Ishii, Koki Maeda, Kazuki Hanauchi, Emika Asechi, Defan Setyawan, Takeshi Niki, Kenji Nakashima, Hirofumi Kurita, Rika Numano, and Takayuki Shibata

Numerical simulation of electric field and plasma membrane lysis

Fig A shows the domain of calculation, governing equations, and boundary conditions for the numerical simulation. (A) Geometry of the microchannel with cell. The total number of elements in the calculations is 274050. (B) Governing equations with their initial and boundary conditions of the lysis model, are the current conservation equation and the asymptotic Smoluchowski equation. (C) In the cell membrane boundary condition equation, J is the current flux through the cell membrane. J(t) consists of the conducting and dielectric currents in the membrane itself and J_{ep}(t). Jep(t) is the current flux through the membrane due to pore formation. A is a coefficient that describes the shape of the membrane (Pucihar, et al., 2009).

A

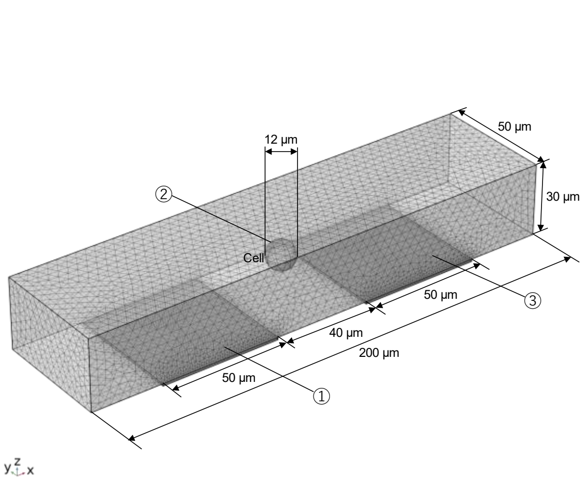

B Electrical membrane lysis (τ~ 1 µs)

i) Current conservation equation	
$$\frac{\partial \rho}{\partial t} + \nabla \cdot \left(\sigma + \varepsilon_0 \varepsilon_r \frac{\partial}{\partial t}\right) \nabla \Phi = 0$$	
BCs	① $\Phi = 0$ ② Continuity of current ③ $\Phi = 10$ V
ICs	Φ_o(x,y,z) = 0
ii) Asymptotic Smoluchowski equation (ASE)	
$$\frac{dN(t)}{dt} = \alpha e^{(V_m/V_{ep})^2} \left\{1 - \frac{N(t)}{N_0} e^{-q(V_m/V_{ep})^2}\right\}$$	
ICs	$N(0) = 0$

C Continuity of current on membrane

i) Boundary conditional equation for cell membrane
$$\mathbf{n} \cdot J = \frac{1}{d}\left(\sigma + \varepsilon_0 \varepsilon_r \frac{\partial}{\partial t}\right) V_m$$
ii) Current flux through the cell membrane
$$J(t) = \frac{\sigma_m(\Phi_i - \Phi_o)}{d} + \frac{\varepsilon_0 \varepsilon_m}{d} \frac{\partial(\Phi_i - \Phi_o)}{\partial t} + J_{ep}(t)$$
iii) Due to pores formation
$$J_{ep}(t) = i_{ep}(t) N(t) = (\Phi_i - \Phi_o) \sigma_p \pi r_p^2 \frac{A}{d} N(t)$$

BCs : Boundary conditions ICs : Initial conditiions

Fig A. Settings of numerical calculation of electric field and plasma membrane lysis. (A) Calculation space imitating the microchannel, electrodes, and a cell. (B) Equations to calculate electric field and plasma membrane lysis. (C) Equations to apply the continuity of current on membrane.

Table A. Parameters of numerical calculations.

Symbol/ abbreviation	Parameter	Value
R	Cell diameter	12 μm (measured Value)
d	Membrane thickness	7 nm (C.P. Jen et al., 2009)
σ_{PBS}	Conductivity of medium	1.38 S/m (measured Value)
ε_{PBS}	Relative permittivity of medium	78 (T. Sun et al., 2010)
σ_m	Membrane conductivity	1.0×10^{-6} S/m (C.P. Jen et al., 2009)
ε_m	Membrane relative permittivity	8 (C.P. Jen et al., 2009)
σ_{cyt}	Relative permittivity of cytoplasm	0.8 S/m (C.P. Jen et al., 2009)
ε_{cyt}	Cytoplasmic permittivity	50 (C.P. Jen et al., 2009)
ε_o	Dielectric permittivity of vacuum	8.854×10^{-12} F/m
V_{ep}	Voltage of electroporation	170 mV (Pucihar et al., 2009)
α	Creation rate coefficient	1×10^9 $1/m^2s$ (Krassowska & Filev, 2007)
N_0	Equilibrium pore density	1.5×10^9 $1/m^2$ (Krassowska & Filev, 2007)
q	Pores creation rate	2.46
r_p	Pore radius	0.8 nm
n	Relative entrance length of pore	0.15 (Krassowska & Filev, 2007)
σ_p	Conductivity of pores	0.16 S/m
w_o	Energy barrier within pore	2.65 (Krassowska & Filev, 2007)
F	Faraday constant	9.65×10-4 C/mol
R	Gas constant	8314 J/(mol・K)
T	Absolute temperature	298 K
E	Electric field strength	100 kV/m

Fabrication

Fig B shows the fabrication process. Microchannels and electrodes were fabricated in different processes and then bonded to each other.

To form the microchannel, soft lithography was performed. Si substrate (dummy wafer, e-Prize Co.) was washed with isopropyl alcohol and acetone and dried by blowing N_2. Photoresist SU-8 3050 (Nippon Kayaku Co.) was spin-coated onto the substrate using the following spin speed: slope (5 s) at 500 rpm, followed by slope (5 s) at 3000 rpm for 30 s, and finally slope (10 s). The substrate was pre-baked ona hotplate with the following temperature profile: 65 °C for 2 min, 95 °C for 15 min, and 65 °C for 2 min, and then allowed to cool for over 5 min.

UV exposure was performed on a double-sided mask aligner (PEM-800, Union Optical co.). Film masks (UnoGiken Co.), designed by us, were used as photomasks. ~230 mJ/cm^2 for the i line was exposed to the photoresist on the substrate. After exposure, post-baking was performed on a hotplate with 65 °C (2 min), 95 °C (5 min), and 65 °C (2 min). Non-exposed part of SU-8 was removed by 10 min dipping in 2-Methoxy-1-Methylethyl Acetate (130-10505, Fujifilm Wako Co.). After washing with isopropyl alcohol and acetone, and drying with N2 blowing, the substrate was exposed to the vapor of trichloro

(1H,1H,2H,2H-perfluorooctyl) silane (448931, Fujifilm Wako Co.) in a vacuum desiccator to improve removal of PDMS. Fabricated substrate was put in the plastic dish, then PDMS (SILPOT 184 W/C, DuPont Toray Specialty Materials K.K.) was poured with a thickness of ~5 mm and stored at room temperature for >3 days for curing. After curing, they are cut with a cutter and peered off manually. The inlet and outlet hall are punched with a biopsy punch.

Electrodes on glass wafers were formed by a lift-off process. At first, a glass substrate (AS-4, Toshin Riko Co.) was subjected to heated sulfuric acid and hydrogen peroxide. After drying with N2 blowing and heating on a hotplate with 140 °C for over 5 min, OAP (Tokyo Ohka Kogyo Co.) was spin-coated with the following profile: slope (5 s) at 500 rpm, followed by slope (5 s) at 4000 rpm for 20 seconds, and finally slope (10 s). The substrate was then baked at 140 °C for 5 min to improve the resist adhesion. Photoresist OFPR-8600 (Tokyo Ohka Kogyo Co.) was spin-coated using the profile: slope (5 s) at 500 rpm for 20 seconds, followed by slope (5 s) at 4000 rpm for 20 seconds, and slope (10 s) and baked with 110°C for 90 s. ~250 mJ/cm^2 for the g line is exposed to the substrate. After exposure, the pattern was developed by dipping to NMD3 (Tokyo Ohka Kogyo Co.) for ~90 s. After washing with pure water, the substrate surface was plasma-treated for 20 s with air and a power setting of 150 W (JPA-300, J-Science Lab Co.). Ti and Pt were deposited by sputtering with L-250S-FH (Canon Anelva Co.). After deposition, the substrate was dipped in the acetone. Photo resist is melted and metal on the resist is removed. Finally, the pattern is checked with a microscope, and the remaining unwanted metal was removed.

PDMS microchannel and glass substrate with electrodes are plasma treated for 60 s with air and a power setting of 150 W (JPA-300, J-Science Lab Co.). After treatment, two materials were attached manually and heated on a hotplate at 80 °C for >15 min.

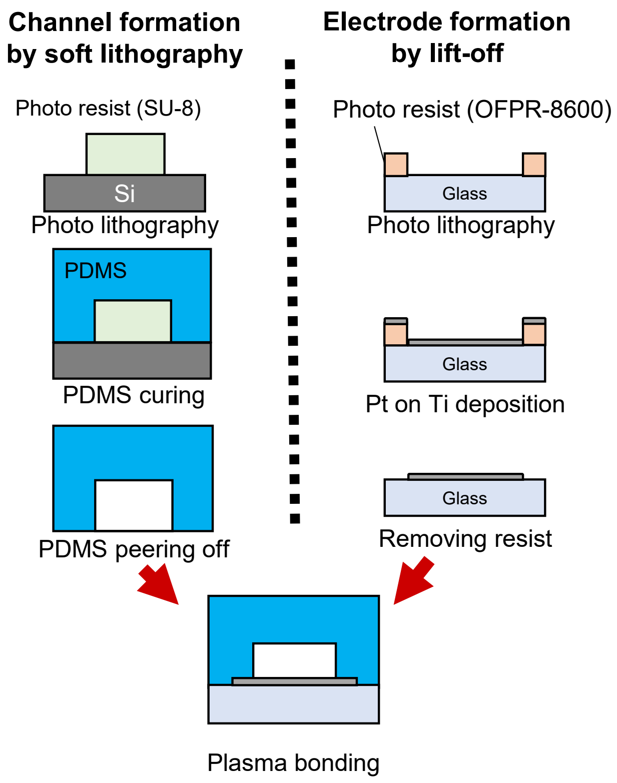

Fig B. Schematic of fabrication method.

Channel shape

Microscopy of the microchannel is shown in **Fig. C**. Cross-sectional shape of the channel is trapezoid. Contacting electrode substrate is narrow. Channel width is measured in the top view. The edge of the channel looks like a double line because the top and bottom of the channel are different because of the cross-sectional shape. Inner and outer width were measured and average value was used as w_1 value.

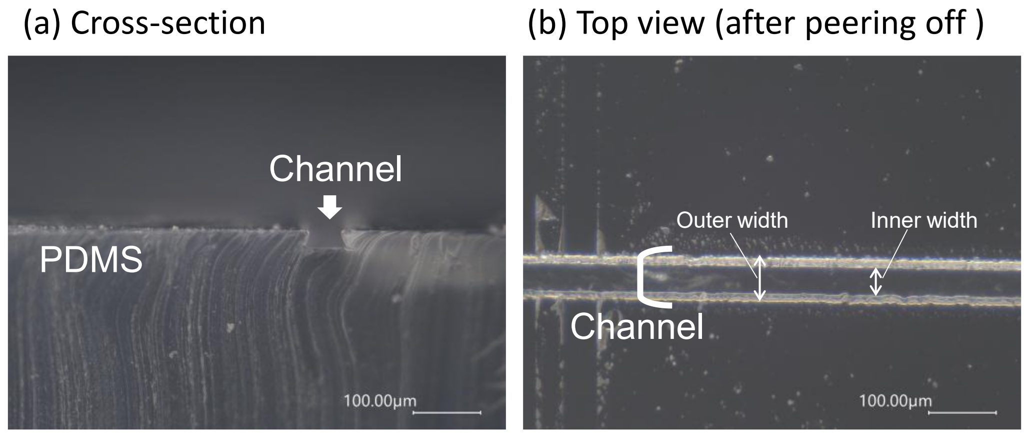

Fig C. Microscopic images of channel. (a) Cross-section of PDMS. (b) Top view of microchannel.

Cell counting

Fig D shows an example of the images taken at 4x magnification, which is used for cell counting.

Image J processing is as below:

1. Run “Substract Background” with the value of “Rolling” of 150.

2. Run “Multiply” with the value of 5

3. Run “Find Maxima” with the arbitral value of prominence. (**Fig. E**)

The optimal value of the prominence sensitively depends on the focus condition. The value was determined manually by examining the image.

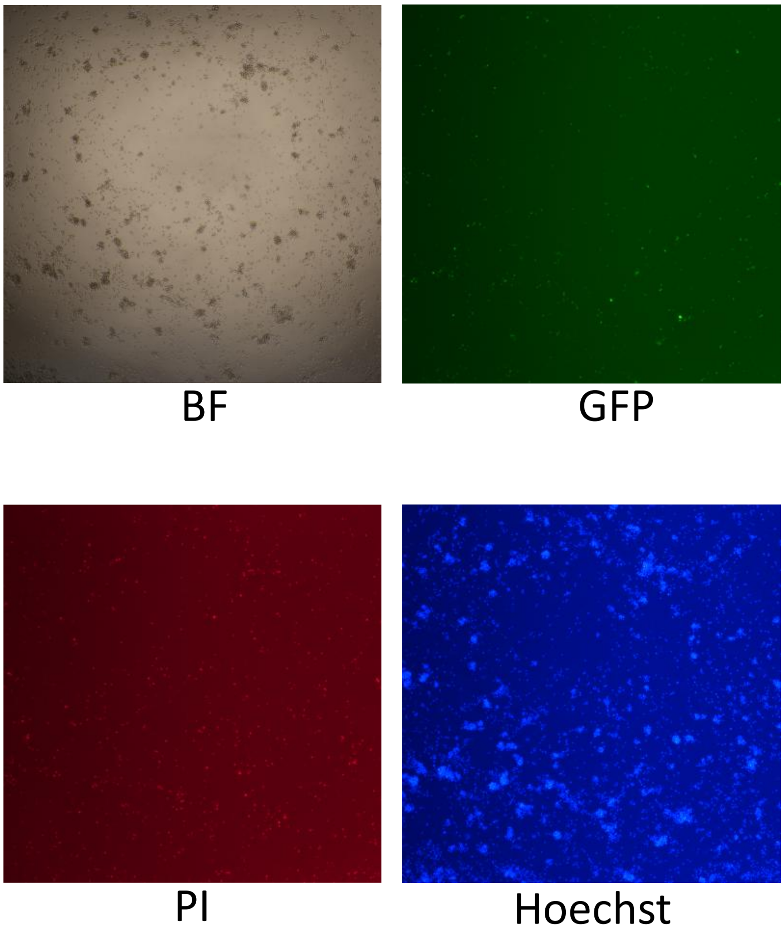

Fig D. An example of fluorescent microscopic images of LCL taken at 4x magnification.

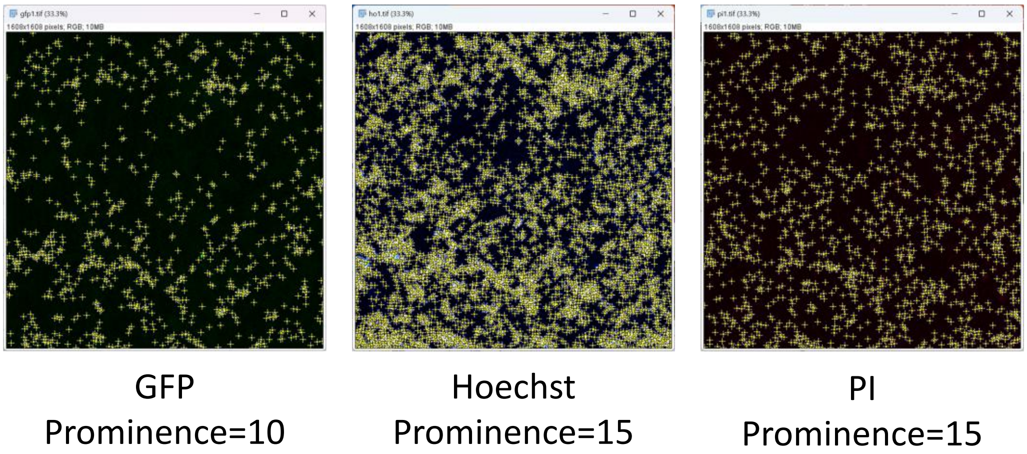

Fig E. Example of cell counting results in Image J. Yellow points show the location of cells.

Data table

Table B. Fluorescent counting results of negative control (Cells and plasmid are mixed, but not installed to the microchannel.)

Sample number	$n_{\mathrm{PI,NC}}$	$n_{\mathrm{GFP,NC}}$	$n_{\mathrm{Hoechest,NC}}$	$n_{\mathrm{PI,NC}}/n_{\mathrm{Hoechest,NC}}$
Ⅰ	1354	5	9580	0.1413
Ⅱ	2099	0	11031	0.1903
Ⅲ	1029	0	7229	0.1423
Ⅳ	1025	0	6856	0.1495
Ⅴ	1252	0	4343	0.2883
Ⅵ	2568	0	9325	0.2754
Ⅶ	3009	3	12570	0.2394
Ⅷ	2568	2	7219	0.3557
Ⅸ	1371	0	4330	0.3166
Ⅹ	1231	0	9708	0.1268
Ⅺ	1907	1	9170	0.2080

Table C. Fluorescent counting results of triangle wave and 10V conditions with several pneumatic pressures. NC numbers correspond to sample number of Table B.

NC number	Applied Voltage V_{in}	Pressure kPa	Velocity mm/s	n_{PI}	n_{GFP}	n_{Hoechest}	GFP expression rate $n_{\mathrm{GFP}}/(n_{\mathrm{Hoechest}}-n_{\mathrm{PI}})$	Cell viability $1-(n_{\mathrm{PI}}/n_{\mathrm{Hoechest}}-n_{\mathrm{PI,NC}}/n_{\mathrm{Hoechest,NC}})$
Ⅰ	10	5	25.4	3518	1217	9453	0.2051	0.7312
Ⅰ	10	7.5	36.6	2197	2097	6976	0.4388	0.7978
Ⅺ	10	7.5	45.0	1907	1323	5822	0.3379	0.8490
Ⅲ	10	7.5	46.9	813	1005	2917	0.4777	0.8410
Ⅰ	10	10	17.5	1829	1016	6593	0.2133	0.8415
Ⅲ	10	10	48.7	667	777	2731	0.3765	0.8812
Ⅶ	10	10	52.2	1762	1002	4524	0.3628	0.8027
Ⅸ	10	10	57.6	1910	1387	4587	0.5181	0.8540
Ⅵ	10	10	60.9	1896	399	3251	0.2945	0.5752
Ⅹ	10	10	63.4	1199	1570	5144	0.3980	0.8783
Ⅷ	10	10	64.6	3084	182	4926	0.0988	0.5804
Ⅺ	10	10	64.6	1472	761	4590	0.2441	0.8577
Ⅲ	10	15	59.8	665	657	2388	0.3813	0.8413
Ⅺ	10	15	108.1	1419	404	3812	0.1688	0.7926
Ⅰ	10	15	109.7	3025	1539	7775	0.3240	0.7115
Ⅲ	10	20	123.8	364	123	1373	0.1219	0.8569
Ⅰ	10	20	165.2	655	174	2240	0.1098	0.8241
Ⅺ	10	20	172.7	1138	740	5105	0.1865	0.9811
Ⅲ	10	30	196.6	397	226	1589	0.1896	0.8747
Ⅺ	10	30	262.5	1404	221	4558	0.0701	0.8737
Ⅰ	10	40	309.7	1275	500	5148	0.1291	0.8762

Table D. Fluorescent counting results of sinusoidal wave and 10V conditions with several pneumatic pressures. NC numbers correspond to sample number of Table B.

NC number	Applied Voltage V_{in}	Pressure kPa	Velocity mm/s	n_{PI}	n_{GFP}	$n_{Hoechest}$	GFP expression rate $n_{GFP}/(n_{Hoechest}-n_{PI})$	Cell viability $1-(n_{PI}/n_{Hoechest}-n_{PI,NC}/n_{Hoechest,NC})$
III	10	5	25.7	3917	636	5177	0.5048	0.2838
XI	10	7.5	50.2	1839	1933	7116	0.3663	0.9363
III	10	10	52.4	941	1774	4941	0.4435	0.9439
IV	10	10	58.5	1071	111	1264	0.5751	0.1795
VI	10	10	70.3	1896	371	2709	0.4563	0.4142
VII	10	10	58.7	2145	534	4143	0.2673	0.6340
IX	10	10	58.0	5189	1117	7072	0.5932	0.3896
XI	10	10	72.3	2823	2408	8336	0.4368	0.8350
III	10	15	97.3	782	373	2537	0.2125	0.8066
XI	10	15	112.1	1384	595	4163	0.2141	0.8428
III	10	20	108.3	1430	558	3981	0.2187	0.7471
IV	10	20	138.5	1352	1199	4100	0.4363	0.7881
XI	10	20	155.5	2773	2324	8953	0.3761	0.8715
III	10	30	136.1	562	906	3739	0.2852	0.9907
XI	10	30	243.3	1905	918	6237	0.2119	0.8769

Table E. Fluorescent counting results of triangle wave and 10 kPa conditions with several applied voltages. NC numbers correspond to sample number of Table B.

NC number	Applied Voltage V_{in}	Pressure kPa	Velocity mm/s	n_{PI}	n_{GFP}	$n_{Hoechest}$	GFP expression rate $n_{GFP}/(n_{Hoechest}-n_{PI})$	Cell viability $1-(n_{PI}/n_{Hoechest}-n_{PI,NC}/n_{Hoechest,NC})$
III	0.0	10	No data	1166	0	11612	0.0000	1.0489
I	0.0	10	69.9	1716	0	16380	0.0000	1.0426
II	0.0	10	84.9	881	0	6751	0.0000	1.0298
VI	0.0	10	62.7	2028	0	7316	0.0000	0.9975
VII	0.0	10	63.2	582	0	3285	0.0000	1.0818
VIII	0.0	10	75.9	2079	5	4000	0.0026	0.7454
IX	0.0	10	76.2	1374	0	3208	0.0000	0.8366
X	0.0	10	73.7	1431	0	9812	0.0000	0.9782
XI	0.0	10	70.2	1770	4	9920	0.0005	1.0373
VI	2.5	10	75.4	1767	1	6520	0.0002	1.0060
VII	2.5	10	54.7	1286	0	4392	0.0000	0.9298
VIII	2.5	10	76.5	2760	0	5764	0.0000	0.8089
IX	2.5	10	76.6	1897	1	5470	0.0003	0.9558
X	2.5	10	72.2	1246	0	8303	0.0000	0.9734
VI	5.0	10	76.2	1527	1	4668	0.0003	0.9286
VII	5.0	10	58.7	521	1	1005	0.0021	0.6332
VIII	5.0	10	72.3	3912	21	6841	0.0072	0.6646
IX	5.0	10	77.1	1301	1	2520	0.0008	0.7079
X	5.0	10	59.3	1313	8	7499	0.0013	0.9447
VI	7.5	10	64.6	1957	592	6065	0.1441	0.9347
VII	7.5	10	56.1	1848	355	4474	0.1352	0.7717
VIII	7.5	10	66.3	4643	665	8603	0.1679	0.7145
IX	7.5	10	42.3	1836	1348	7636	0.2324	1.1115
X	7.5	10	43.2	1551	880	7501	0.1479	0.9084
III	10.0	10	48.7	667	777	2731	0.3765	0.8812
I	10.0	10	17.5	1829	1016	6593	0.2133	0.8415
VI	10.0	10	60.9	1896	399	3251	0.2945	0.5752
VII	10.0	10	52.2	1762	1002	4524	0.3628	0.8027
VIII	10.0	10	64.6	3084	182	4926	0.0988	0.5804
IX	10.0	10	57.6	1910	1387	4587	0.5181	0.8540
X	10.0	10	63.4	1199	1570	5144	0.3980	0.8783
XI	10.0	10	64.6	1472	761	4590	0.2441	0.8577
II	12.5	10	88.8	3170	10	4422	0.0080	0.3353
VI	12.5	10	58.8	4007	866	6939	0.2954	0.5831
VII	12.5	10	No data	2936	395	4682	0.2262	0.4903
VIII	12.5	10	62.6	3303	76	4158	0.0889	0.3192
IX	12.5	10	50.1	2806	140	3805	0.1401	0.3842
X	12.5	10	65.7	1691	1279	4589	0.4413	0.7232

Table F. Fluorescent counting results of sinusoidal wave and 10 kPa conditions with several applied voltages. NC numbers correspond to the sample number of Table B.

NC number	Applied Voltage V_{in}	Pressure kPa	Velocity mm/s	n_{PI}	n_{GFP}	$n_{Hoechest}$	GFP expression rate $n_{GFP}/(n_{Hoechest}-n_{PI})$	Cell viability $1-(n_{PI}/n_{Hoechest}-n_{PI,NC}/n_{Hoechest,NC})$
III	0.0	10	No data	1166	0	11612	0.0000	1.0489
VI	0.0	10	62.7	2028	0	7316	0.0000	0.9975
IV	0.0	10	62.4	966	0	5428	0.0000	0.9665
VII	0.0	10	63.2	582	0	3285	0.0000	1.0818
VIII	0.0	10	75.9	2079	5	4000	0.0026	0.7454
III	0.0	10	65.5	1172	0	2501	0.0000	0.7466
VIII	0.0	10	76.2	1374	0	3208	0.0000	0.8366
X	0.0	10	73.7	1431	0	9812	0.0000	0.9782
XI	0.0	10	70.2	1770	4	9920	0.0005	1.0373
VI	2.5	10	65.5	2492	3	7597	0.0006	0.9274
VII	2.5	10	63.7	1561	8	7476	0.0014	1.0402
VIII	2.5	10	73.6	3133	1	6433	0.0003	0.7962
IX	2.5	10	66.6	1993	3	7707	0.0005	1.0849
X	2.5	10	69.6	1847	7	10547	0.0008	0.9447
VI	5.0	10	58.9	2954	461	10846	0.0584	1.0042
VII	5.0	10	61.2	1059	30	4625	0.0084	1.0137
VIII	5.0	10	72.7	3554	109	7079	0.0309	0.7729
IX	5.0	10	65.5	1513	47	5252	0.0126	1.0418
X	5.0	10	53.0	1648	111	8005	0.0175	0.9094
VI	7.5	10	62.7	2247	513	6495	0.1208	0.9026
VII	7.5	10	60.7	1186	652	4440	0.2004	0.9635
VIII	7.5	10	65.6	4187	523	6337	0.2433	0.5266
IX	7.5	10	63.1	2897	1743	6803	0.4462	0.8402
X	7.5	10	20.4	1492	1005	6022	0.2219	0.8615
III	10.0	10	52.4	941	1774	4941	0.4435	0.9439
IV	10.0	10	58.5	1071	111	1264	0.5751	0.1795
VI	10.0	10	70.3	1896	371	2709	0.4563	0.4142
VII	10.0	10	58.7	2145	534	4143	0.2673	0.6340
VIII	10.0	10	71.3	4454	182	6131	0.1085	0.4246
IX	10.0	10	58.0	5189	1117	7072	0.5932	0.3896
X	10.0	10	65.1	1331	847	3798	0.3433	0.7439
XI	10.0	10	50.2	1839	1933	7116	0.3663	0.9363
IV	12.5	10	66.7	2605	47	2798	0.2435	0.0811
VI	12.5	10	63.5	1982	41	2703	0.0569	0.3681
VII	12.5	10	58.8	3314	29	3823	0.0570	0.1750
VIII	12.5	10	67.4	2680	21	3755	0.0195	0.4444
IX	12.5	10	46.6	4147	24	4367	0.1091	0.0737
X	12.5	10	64.2	1470	119	2080	0.1951	0.3359

Table G. Alkaline-phosphatase-stained colony counting results for iPSCs reprogramming evaluation.

Sample	Applied voltage V_{in} V	Waveform	Pressure kPa	Velocity mm/s	The number of colony
A	10	Triangle	10	59.4615	45
B	10	Triangle	10	61.03	38
B	10	Triangle	10	60.255	16
A	10	Sinusoidal	10	62.07	33
B	10	Sinusoidal	10	62.32	45
B	10	Sinusoidal	10	57.76	13
A	12.5	Triangle	10	41.906	52
B	12.5	Triangle	10	60.516	63
B	12.5	Triangle	10	53.002	57
A	12.5	Sinusoidal	10	60.44	22
B	12.5	Sinusoidal	10	60.516	9
B	12.5	Sinusoidal	10	56	1
A	12.5	Sinusoidal	20	133.232	56
B	12.5	Sinusoidal	20	149.056	48
